# The Calgary student run clinic in context: a mixed-methods case study

**Published:** 2019-07-24

**Authors:** Danielle Smith, Sharanya Ramesh, Matthew Smith, Ashley Jensen, Rachel Ellaway

**Affiliations:** 1 Cumming School of Medicine, University of Calgary, Alberta, Canada

## Abstract

**Background:**

Student Run Clinics (SRCs) provide students with clinical education while caring for underserved populations. While much of the research on SRCs comes from the USA, SRCs in other contexts need to be appraised in the context of the systems they interact with. This study explored how stakeholders in the University of Calgary’s SRC perceived its purpose and beneficiaries with respect to patients, students, undergraduate medical education, and its intersections within the healthcare system in Calgary.

**Methods:**

Data came from the SRC’s EMR and stakeholder interviews at the Inn from the Cold (IFTC) shelter. Qualitative data were analyzed using standard grounded theory techniques.

**Results:**

There were 13 interviews - seven with student clinicians and six with preceptors and other stakeholders. Interviews highlighted the uncertainty of the SRCs role. Majority of participants saw the SRC as facilitating further access to other healthcare services, while some commented on its primarily education-focused role. Major limitations in the SRC’s scope of care and its integration with other services were identified.

**Conclusion:**

SRCs need to consider their accountabilities, both educational and healthcare-focused at individual and organization levels, in order to function as responsible healthcare providers in Calgary.

## Introduction

Student Run Clinics (SRCs) have been set up across North America as a way of providing students with additional clinical education and leadership experience, while concurrently providing services to underserved populations.^1^ SRCs vary in their organizational structure and the services they offer, but they are typically operated and managed by medical students with oversight from practicing physicians,^1^ which means that students can learn from their involvement in the administration and coordination of SRC services as well as from providing supervised clinical care.^2^ Although SRCs are primarily educational initiatives,^3^ they are also expected to provide valuable healthcare services to patients, a balance that can prove challenging.^4^

In addition to educational and healthcare considerations, SRCs also need to consider their functions within the broader healthcare and educational contexts in which they function. An SRC’s operating context includes local healthcare providers that SRC patients may be referred to, as well as the parent MD program educating the SRC executives and clinicians. The local healthcare context can have a major influence on how SRCs are configured, with SRCs in the United States (US) tending to focus on low-income patients without health insurance.^3^ While there are similarities between SRCs in different contexts, the original US model of an SRC may not necessarily translate to other countries such as Canada.^5^ A previous study into the University of Calgary’s SRC noted patient satisfaction with the quality of care provided by the SRC,^6^ however, the structure of the Calgary SRC has changed dramatically in size and location since the publication of this study.

From a medical school perspective, SRCs typically function as an extra-curricular option rather than as a part of the core curriculum. This means that there may be minimal oversight from faculty (unless functioning as supervising clinicians) and little alignment with participating students’ studies.

More importantly, there is a systemic ethical question regarding SRCs relationships with their patients and the broader populations they represent.^7^ On one hand, the argument has been made that SRCs are intrinsically altruistic in that they care for otherwise underserved populations.^3^ An alternative argument could be made that SRCs potentially exploit the underserved to gain additional educational experience and in return provide sub-optimal care. If marginalized groups have little choice in the services they can access, they may be willing to accept a lower standard of care than the mainstream population might accept in order to access available services. To that end, the role of an SRC as a service provider needs to be well aligned with the local environment within which it is situated in order to ensure that the underserved patients it sees are not further disadvantaged by their use of SRC services.

This paper describes a study of the role of the Calgary SRC within its local environment with respect to patient needs, services, and the intersection with its local healthcare system. In doing so we sought to identify ways in which the University of Calgary SRC might be better structured to fit within its local healthcare environment.

## Methods

We designed the study as part of a larger mixed methods case study into the Calgary SRC. The case study was bound in place to the University of Calgary SRC and in time to 2016.^8^ Case study is an eclectic methodology, involving the collection of a variety of data to provide a “thick” description of the topics of interest.

### Study context

The Calgary SRC was established in 2011 at a local homeless shelter by medical students working with a local clinical preceptor. It expanded both in size and services to the point that it was providing care at three sites in downtown Calgary at the time of the study in 2016 – see Box 1 for details. The University of Calgary runs a three-year MD program, and students’ 12-month involvement in the SRC corresponds with the part of the first and second year of their studies. A total of 36 students participated in the SRC in the 2015-2016 year (approximately 23% of the class), of which 28 undertook a clinician role and eight were in executive (leadership and/or administration) roles. There was a strong student interest in participating in the SRC with roughly half of the class applying to participate each year. Student applicants were selected to participate as clinicians via lottery, while SRC executive positions were selected via interviews with past year executives. Most of the student’s participation in the SRC took place at the Inn from The Cold (IFTC) site.

Box 1 **Calgary, University of Calgary SRC Locations, and other Healthcare Services for Homeless in Calgary**

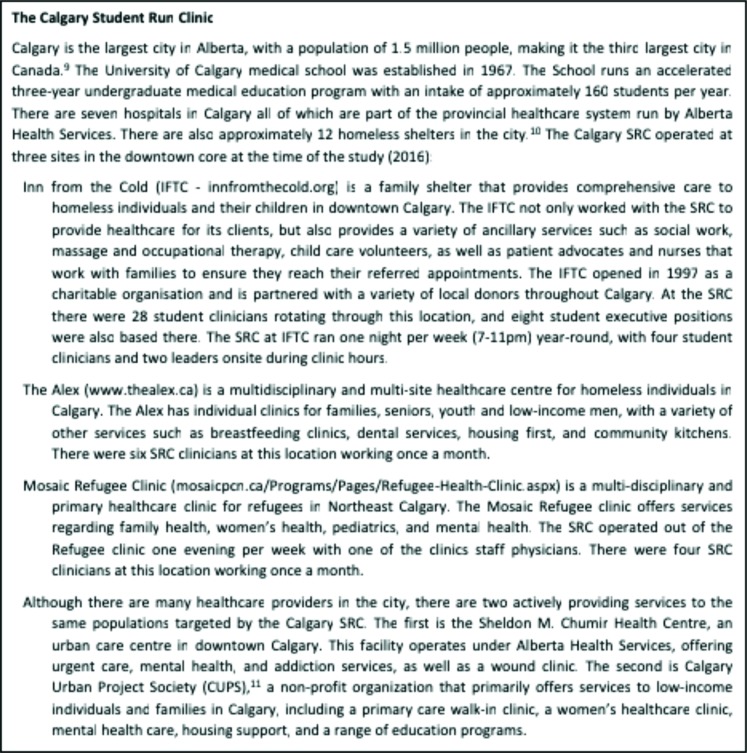

### Sample

A convenience sample of current SRC students at the IFTC, preceptors, and others involved in running the SRC was used to give a range of different perspectives. Numbers of participants in each category were bound by the total number in the study population and by a willingness to participate in the study.

### Data collection

The study drew on two data sources: aggregated patient data from the SRC’s Electronic Medical Record (EMR) and interviews with students, and with preceptors, education leaders, and staff associated with the SRC. The semi-structured interview scripts were developed iteratively based on discussions among the study team and on piloting the instrument with the student members of the team – see Appendices A and B. Interviews were conducted by study author RE who had no prior relationship with any study participants. The interviews were audio- recorded. The standalone electronic medical record (EMR) (Telus Health PS-Suite) was queried between January 2014 and October 2016 for patient sex, patient age, total appointments, and pediatric assessments completed between January 2014 and October 2016.

### Analysis

Descriptive statistics were generated for all quantitative data collected from the EMR. Qualitative data from the interview transcripts were analyzed by all study authors (DS, SR, MS, AJ, RE) until transcript coding reached data saturation. Themes related to the study question were developed using constructivist grounded theory techniques (line-by-line coding, memo-ing, axial coding).^12^ Note that, while we used techniques from grounded theory, this was not a grounded theory study.^13^

### Research team

This was a student-initiated study involving two SRC executive members in 2015-2016 (MS, AJ) and their successors in 2016-2017 (DS, SR), guided and overseen by an experienced medical education researcher.

### Ethics

The study was approved by the University of Calgary’s Conjoint Health Research Ethics Board (ID: REB16- 0110).

## Results

Our analyses from the broader study identified several themes and topics. In this paper we report on those findings that are relevant to our stated research questions regarding the nature of the services offered, and how the SRC functioned as part of the local healthcare system. We first report on the nature of the services offered and then on how the SRC and its services fit in to the broader local healthcare landscape.

### The activities of the SRC

The SRC at the IFTC had seen 760 patients across 1152 appointments over 145 clinic sessions between 2014 and 2016. The IFTC is a short-term low-barrier family shelter and 59% of patients seen there were under the age of 18. In late 2015, the SRC began a twice monthly pediatric assessment clinic to complete developmental screening for children aged 0 to 5 years staying at the shelter. Since the addition of these sessions, 106 screenings had been completed by the end of 2016 and 30% of these patients had been referred to other facilities for further workup and treatment.

The SRC at the IFTC frequently saw patients with primary care related illnesses, such as the common cold, diarrhea, infection, mental health complaints, and musculoskeletal diseases. Student clinicians at IFTC referred patients to the nearby Sheldon M. Chumir Health Centre if urgent care services were required, or to the Calgary Urban Project Society (CUPS) for dental health services and other long-term primary healthcare needs.

While the SRC at the IFTC was a “pop-up clinic” (running once or twice a week, except when students had exams), the Alex Bus and Refugee Clinic sites were run as daily clinics independent of SRC activity. SRC student clinicians were placed at these two smaller sites, but SRC executive students were not. The SRC operated out of the Alex Bus once a month, assisting with after-hours services offered by the Community Health Bus, a mobile clinic that operated twice weekly. At the Refugee Clinic, student clinicians assisted practicing physicians once a month with clinical services either on weekends or in the evenings. In this sense, the SRC at the Alex and Refugee Clinic were considered satellite student placements with the IFTC being the central SRC operating site. There were six student clinicians placed at the Alex bus and four students at the refugee clinic, while the IFTC had 18 students total with four clinicians and two leadership students working at the clinic in any given week.

### Stakeholder interviews

We conducted 13 interviews, seven with student clinicians and executives from IFTC, and six with IFTC preceptors and other stakeholders (including Undergraduate Medical Education [UME] leaders and IFTC staff). We have organized our findings from the thematic analysis of the interview transcripts to focus on the role of student run clinics in the local Calgary healthcare system.

### The role of the SRC

We found differing beliefs and opinions regarding what the role of the SRC was. Many students saw the primary role of the Calgary SRC as a gateway for marginalized individuals to access healthcare services in Calgary. For instance:

We give healthcare to people that might not be able to access it otherwise and we put them in contact with people if we can't help them … we're that starting point, that home base for them. (Student clinician, interview A)

Others saw the SRC as a way of getting additional clinical learning opportunities. However, many students also noted the ambiguous and potentially conflicting roles of the SRC as a teaching venue and as a healthcare centre for marginalized individuals:

I think we have really good intentions and everyone's really enthusiastic, but I still sometimes think that we are more focused on our own learning than we are on the … recipients of our work. (Student executive, interview B)

I would imagine most people have not been inside a homeless shelter or have found themselves in a place like that, so I think learning wise that in itself is a learning experience, and I hope that this is part of a broader learning. (IFTC staff, interview C)

Both preceptors and students identified a variety of limitations of the SRC that potentially contributed to this lack of clarity regarding its role, including a lack of resources, time constraints, students’ level of training, and unclear teaching objectives. Another recurring issue was that students served no more than a one-year term with the Calgary SRC, which meant that there was a completely different group of students involved in organizing and running the SRC from one year to the next. It became clear that the SRC had operated with more of an aspirational healthcare focus than one based on clear service objectives and had been driven by student and preceptor interest rather than a formal assessment of client needs. Although there were structured handovers between student leaders during the transition to new leadership each year, the healthcare focus had become diffused over time.

### Quality of care provided by the SRC

Both students and preceptors identified barriers to optimal care for patients within the SRC, especially with regards to accessing allied healthcare services, as well as limited resources for procedures. One of the SRC preceptors observed:

[…] the medicine part of it is virtually the same, it’s just here at the clinic we have fewer resources. So, for example if someone had an abscess, in the emergency room I’d usually open that up, cut it out, send it to Pathology and be done, versus someone that might have the same presentation here at the clinic. We just don’t have the ability to do that. (Physician, interview D)

Both preceptors and students identified areas in which the SRC may potentially provide suboptimal care. One preceptor expressed their concerns as follows:

I don't think we're always giving them the best care, and that's because we don't have the facilities available, we don't have you know the clinicians go in and do their history which is not necessarily [a family medicine] history, and you leave the room with like 10 more issues that you walked in with and then, you know, and you're in a time crunch. (Physician, interview E)

These concerns, expressed both by students and preceptors at the IFTC site, were consistent and recurring. While comparable data from the other SRC sites (Alex bus, Refugee Clinic) was not forthcoming, informal student feedback from these sites indicated that the limitations on services available through the IFTC were less of an issue at these other locations. This could be due to the more established way in which these satellite clinics were run, which may have resulted in less ambiguity over patient benefits, and the role of student clinicians in providing patient care.

[…] of the things that does - unfortunately is a barrier for some people is how late it runs and how late it starts because we do have little kids who are going to bed and I think the other challenge is sometimes how long the interviews are. (IFTC staff, interview C)

### Integration within the local healthcare system

Although some referrals were made from the SRC to other local health providers, the SRC was not an Alberta Health Services (AHS) run facility and as such it was not an integrated part of the local healthcare system.

[…] it’s not a formal clinic per se … it follows the rules of the Alberta Health Services … are we providing, you know, the best care ever? Probably not, because we’re, we’re kind of this mobile like pseudo clinic, but are we fulfilling a niche that, a niche some other services are not, I think we are. (Physician, interview F)

The presence of AHS preceptors and the IFTC staff nurse allowed referrals to other services. However, given that the SRC only ran one night per week and that it was very limited in the services it could provide (beyond providing basic health screening, prescriptions, referrals, and consultations), it was not a full clinical unit. Some of the preceptors noted that the tension between the needs of the students and the client population was potentially problematic:

[…] when the students feel that they’re not able to balance school work with seeing patients then they’re more inclined to close the clinic. If it was up to me I would continue to run the clinic, because it’s an expectation of the clients and the staff that we’d be there, but it’s not up to me, it’s very, very much student run. (Physician, interview F)

The ethical problem of running a clinic based on student availability was tempered by a sense that the client population was not abandoned when the SRC did not run: *“[…] there's some excellent resources in downtown Calgary for the underserved population.”* Indeed, there was a lack of clarity as to which patients were already using other healthcare services in the city. For instance, some preceptors noted that they were cautious not to interfere in a patient’s care regime from another provider:

[…] a lot of the patients I was seeing did have family doctors or at least they had been going to someone managing their chronic problem over time. And so … you patch it up and give them a quick refill and make sure everything's okay and then strongly encourage them to see their family doctor again. (Physician, interview E)

However, for those that were not accessing other services then the SRC was more critical in their care, particularly for homeless or immigrant patients:

[…] this is for a lot of people here the first time they've seen any medical staff or a doctor specifically in a very, very long time, so it's pretty vital in that way that this may be their entry back into our medical system … the amount of referrals we do as well connects them just automatically with the community in terms of schedule and specialists and, you know, mental health and all of all of those things. (IFTC staff, interview G)

There was a sense (more from preceptors than students) that the services the SRC offered, should be offered more continuously to accommodate those who needed them most:

[It is challenging] seeing things through to fruition with a population that’s very transient. So, they will see a patient, but they don’t know what’s happened in the follow- up and sometimes [they wonder] did this patient make it to Ophthalmology, what was the diagnosis? … that’s what I keep hearing. (Physician, interview H)

Continuity was also an issue in terms of the transient nature of the population:

[…] once our clients … leave the Inn from the Cold, they no longer are able to access SRC, and so that’s a bit of an inhibiting factor. (Physician, interview H)

Given the complete changeover in the SRC student body each year, we had anticipated that continuity of student participation would be a concern. However, our participants did not seem particularly worried about this.

In summary, although referrals could be made to other services (through supervising physicians), it was not an established part of the local healthcare system. Moreover, the episodic nature of the SRC, the transient nature of the populations it served, and the limited range of services it offered substantially limited the continuity of patient care the SRC was able to provide, which also limited its role within the broader local healthcare system.

### Integration with the local MD program

While we have primarily focused on the healthcare side of the Calgary SRC’s local integration, we should also note that issues were raised with respect to the integration of the SRC with the Calgary MD program. The SRC operates as an extra-curricular student club which is not overseen by the UME, and only a small subset of students participates each year. Moreover, the services used by the patients it saw varied according to individual needs and circumstances. This meant that there was no effective alignment between SRC activities and the MD curriculum, which would sometimes mean that student clinicians were exposed to medical issues they had never seen or been taught. However, students and preceptors worked together to create effective learning environments, and students often felt well- supported:

[…] the people in Student Affairs, they know enough about Student-Run Clinic to know we're student learners, we're in a situation with a superior. And they can give us coaching on what's the best way to move forward and we can give them examples saying, I was in this situation and I didn't sit right, how do I move forward. (Student clinician, interview J)

While the SRC ran autonomously from UME, it did benefit the MD program symbolically in the context of medical school accreditation standards:

[…] in the context of the accreditation … was the need for medical schools to be engaging in service learning where students are learning in an environment and they're giving something back to the community in which they're learning. (UME, interview L)

## Discussion

**Figure 1:**
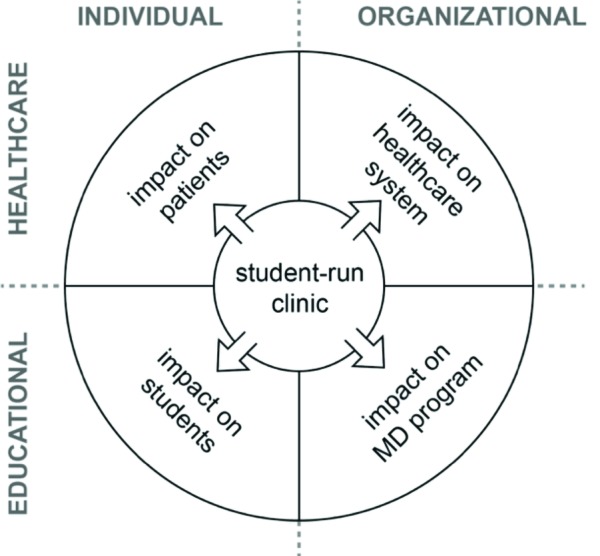
A stakeholder planning and evaluation model for student-run clinics. These align with a grid based on an individual or organizational focus on one axis and an educational or healthcare focus on the other axis. Different SRCs may engage these stakeholders in different ways and to different extents.

This study highlighted the myriad of impacts that the Calgary SRC has on its local environment to further elucidate the purpose of the Calgary SRC and its primary beneficiaries. While the Calgary SRC had been established with a dual education and service goal, its role in meeting the needs of the patient population it served was ambiguous, particularly with respect to those needs that were not met by other providers. This contributed to a broader lack of clarity as to what the primary role of the SRC was and what outcome measures it might use to evaluate whether it was meeting its objectives. To that end, we developed a model from our findings and interpretations of the different dimensions of accountability for SRCs with respect to their local environments. The model was developed iteratively out of our analyses and group discussions and out of group writing. The model focuses on a matrix of accountabilities in terms of their educational or healthcare focus, and whether they apply at the individual or organization level – see Figure 1.

At the individual level, SRCs impact both the patients they serve and the medical students volunteering their time. While the present study identified a perceived education benefit to student participation in the SRC, we also found ambiguity and uncertainty about whether the focus was (or should be) primarily on education or on service. While the tension between the role of the SRC as an education-focused activity and its role as a service provider has been raised by others,^4^ the lack of shared understanding about where the focus of the SRC should be, and how its many responsibilities should be met, contributed to a broader lack of clarity as to what outcome measures it might use to evaluate whether it was meeting its objectives.

Despite this tension there was evidence that the Calgary SRC had benefitted the populations it sought to serve. This reflects findings from other studies that have shown that SRCs can help individuals who have challenges accessing healthcare services due to income difficulties, a lack of affordable childcare, discrimination, and transportation issues.^14,15^ Locating the SRC inside the IFTC potentially addressed some of the barriers to care that homeless families face, and many students saw the role of the SRC as an easily accessible point of healthcare contact for marginalized populations in Calgary. Whether that was actually the case was unclear from our data. However, further studies would be needed to determine the extent to which patients at the SRC are also accessing other health services and whether their use of healthcare services in the SRC differs from other available services in the city.

The relatively novel contribution from this study is the discovery of the extent and ways in which the Calgary SRC did and did not function as a part of the local healthcare system. Our participants noted the current limitations of the SRC in providing services that connected with other providers and that met the long-term needs of the local population. Arguably the biggest gap in services between the SRC and other centers was the limitations in its ability to follow-up with patients, especially given the pop-up nature of the clinic, along with transiency of the populations served at the IFTC. The structural and procedural limitations of a clinic that ran one evening a week at one site and one evening a month at two other sites limited its clinical impact, both in serving the target patient base and in establishing its legitimacy as a clinical service. In addition, the lack of onsite facilities, supplies, and materials that would be needed to provide more comprehensive care further limited the SRC’s ability to serve its patient base.

Clearly an SRC can have multiple accountabilities and potential dimensions of integration. This begs the questions, who is or should be a beneficiary of an SRC, and who is or should be accountable for an SRC? We recommend that those dimensions be made explicit and the accountabilities in each are made a part of the operations and evaluations of SRC programs. Acknowledging the different accountabilities an SRC has is a starting point, but we also need better clarity as to how many and what kinds of patients use SRCs as one provider among many, and how many patients use an SRC as their only access point to the healthcare system. While this would mean expanding the scope of responsibility of SRCs, it could also afford new opportunities for students to better understand the impacts of their patients’ environments on their overall health. At a broader level, this study also raises concerns over the role of “service learning” in medical education (an accreditation requirement in Canadian MD programs) where the balance of benefits and harms, particularly to vulnerable populations, may not be a primary concern.

We acknowledge several limitations within this study. This was an intrinsically idiographic study, both in terms of the small number of participants at only one SRC site (IFTC), as well as the many local idiosyncrasies. However, as a small single site case study our responsibility is to provide a “thick description” of the local context so that generalizability can be assessed by the consumers of the study,^16^ and to that end we propose that the application of our findings is much broader than informing local policies and procedures.

Secondly, while we would have liked to have included patient voices in the study, engaging patients proved very difficult. A patient survey was attempted but there was very a small response number to the initial survey, and ethical limitations on how much persuasion (i.e., with compensation) could be put on them to participate in the study. Similarly, the perspectives of the other healthcare providers might have been useful in order to identify the quantity of patients and the presenting concerns that differ between the SRC and other healthcare locations. Member checking was not used to detail and reflect on the provisional findings from the study, as the logistics of organizing to do so proved prohibitive.

Finally, the generalizability of our findings should be considered in the context that Calgary is one of two three-year MD programs in Canada. This places restrictions on how long our medical students can participate in SRC activities: there is a smaller window of opportunity for U of C students to get involved in the SRC than for students at four-year medical schools. This in turn may mean that our SRC students were less experienced than those students from other schools who volunteer at an SRC past their first year. Furthermore, the nature of the Calgary MD program means that the window of time students can be involved in the Calgary SRC is limited to 12 months, which means that a brand-new group of students is involved each year. This student turn-over has contributed to the diffusion of the SRC’s purpose over time. While this may influence both the range and quality of services offered by the Calgary SRC as compared to SRCs elsewhere in Canada, it was not a goal of the study to make these kinds of comparisons. Similarly, the specifics of the local healthcare system will be unique to Calgary and the extent to which the Calgary SRC is accountable to its unique environment may not be reflected elsewhere.

The findings from this study have informed planning for the Calgary SRC and have already led to several changes in how the clinic functions. For instance, the Calgary SRC is currently in transition to find further locations beyond the IFTC, and primarily functions solely at the Alex bus and Refugee clinic at the time of writing. The SRC executive team are also initiating five-year goals with patient perspectives in mind in order to further focus on the purpose and to serve the stakeholders in the SRC in Calgary

### Conclusion

With this study, we aimed to identify ways in which the Calgary SRC aligned and intersected with its local contexts. While student, patient, and program needs have previously been considered in other studies, SRCs should be also evaluated in the context of their local healthcare systems. On-going changes to the Calgary SRC will aim to bring clarity to the purpose of the SRC among other services currently being provided in Calgary. SRCs should be cautious when adopting models developed in different healthcare contexts and should seek to understand the role of these clinics within their own local environments.

## Appendix A

**Student interview questions**

1. What medical school year are you in?
2. What is your role at the Student Run Clinic?
3. Which site are you located at?
4. Was this your preferred location?
5. Why did you volunteer to work with the Student Run Clinic?
6. What did you hope to gain? Have you changed your perspective from your participation?
7. What have been the benefits to you from being a part of the Student Run Clinic?
8. What have been the drawbacks to you from being part of the Student Run Clinic?
9. What has it been like seeing Student Run Clinic patients?
10. What about seeing patients with a partner?
11. What has it been like working with the Student Run Clinic supervisors?
12. What do you estimate the time commitment of participating in Student Run Clinic has been?
13. Did your experience match your expectations?
14. How prepared did you feel you were for your work at the Student Run Clinic?
15. How clear are you about your role and responsibilities within the Student Run Clinic?
16. What works well for you at the Student Run Clinic?
17. What would you change about the Student Run Clinic?
18. Will participating in the Student Run Clinic make you a better Doctor?
19. Is there anything else you would like to add?

## Appendix B

**Supervisor and support staff questions**

1. What is your role in your day job?
2. What clinical practice settings do you work in?
3. Do you have any academic affiliations with the University?
4. What is your role in the Student Run Clinic?
5. What has been your involvement with the Student Run Clinic? How long? How often?
6. Which site(s) of the Student Run Clinic do you work at?
7. How did you first become involved with Student Run Clinic?
8. What is your perceived benefit from participating in the Student Run Clinic?
9. What are the patient/client benefits from the Student Run Clinic?
10. What capabilities do students need to participate fully in the Student Run Clinic?
11. To what extent do learners come with the required skills to participate?
12. Are you involved in screening learners wanting to participate in the Student Run Clinic?
13. How much teaching do students need in order to participate?
14. How much oversight do students need?
15. To what extent is Student Run Clinic about providing care or about teaching?
16. What risks or challenges to learners do you see in the Student Run Clinic?
17. What risks or challenges to patients do you see in the Student Run Clinic?
18. What risks or challenges to healthcare professionals do you see in the v?
19. How and to what extent does Student Run Clinic fit into the broader healthcare system?
20. What works best in the Student Run Clinic?
21. If you could change anything, what would you change?
22. Do you have any other comments?
